# Can Certain Antihypertensives Prolong the Efficacy of Hyaluronic Acid Injections in Patients with Osteoarthritis of the Knee? Post Hoc Analysis of a Prospective Observational Trial (PRESAGE)

**DOI:** 10.3390/jcm15051935

**Published:** 2026-03-04

**Authors:** Arthur Dollinger, Thomas Lohse, Clara Dolci, Charles Rapp, Charlotte Bourgoin, Anne Lohse, Thierry Conrozier

**Affiliations:** 1 Department of Rheumatology, Hôpital Nord Franche-Comté, 100 Route de Moval, CS 10490, Trevenans, 90015 Belfort, France; arthur.dollinger@chu-montpellier.fr (A.D.); cj.rapp@outlook.fr (C.R.); anne.lohse@hnfc.fr (A.L.); 2 Department of Cardiology, Hôpital de la Croix-Rousse, Hospices Civils de Lyon, 69004 Lyon, France; thomas.lohse@chu-lyon.fr; 3 Unité de Soins Intensifs Cardiologiques (USIC), Hôpital Cardiologique, 69500 Bron, France; clara.dolci@chu-lyon.fr; 4 Clinical Research and Innovation Unit, Hôpital Nord Franche-Comté, 90015 Belfort, France; charlotte.bourgoin@hnfc.fr

**Keywords:** antihypertensives, hyaluronic acid, viscosupplementation, osteoarthritis, predictors, arterial hypertension

## Abstract

**Background:** Arterial hypertension (AH) is a frequent comorbidity in patients with osteoarthritis (OA). Among antihypertensive agents, angiotensin-converting enzyme (ACE) inhibitors, calcium channel blockers (CCBs), angiotensin II receptor blockers (ARBs), and beta-blockers (βBs) have been suggested to influence OA progression and symptomatology. The aim of this study was to assess whether the duration of effectiveness (DE) of viscosupplementation (VS) differs between patients with knee OA who are receiving antihypertensive treatment and those who are not. **Methods:** This post hoc analysis was conducted using data from a cross-sectional clinical trial (ClinicalTrials.gov Identifier: NCT04988698). The study included consecutive patients with knee OA who came for consultation at the Rheumatology Department and had received intra-articular hyaluronic acid injections within the past three years. The primary outcome was DE, self-reported by patients as the number of weeks of symptom relief. Associations between DE and various factors, including demographics, disease duration, radiographic OA severity (Kellgren–Lawrence grade and affected compartments), comorbidities, OA treatment history, antihypertensive therapy, physical activity level, and prior VS sessions, were analyzed using bivariate and multivariate models. **Results:** A total of 105 patients (65 women, 149 treated knees) were included. The mean age was 66.1 ± 13.2 years, and the mean body mass index (BMI) was 27.5 kg/m^2^. Thirty-eight percent of patients were receiving antihypertensive treatment (mean number of agents: 1.9; range: 1–4), including CCBs (n = 15), ACE inhibitors (n = 13), ARBs (n = 7), βBs (n = 6), and diuretics (n = 2). The overall mean DE of VS was 48.2 ± 24.8 weeks, with a trend toward longer DE in hypertensive patients compared to non-hypertensive patients (53.1 ± 31.3 vs. 45.4 ± 19.8 weeks, *p* = 0.06). Bivariate analysis identified significantly longer DE in patients with BMI < 27.5 kg/m^2^ (*p* = 0.002), Kellgren–Lawrence grade < 4 (*p* = 0.008), an active lifestyle (*p* = 0.005), unicompartmental OA (*p* = 0.01), medial tibiofemoral joint space narrowing (*p* = 0.046), and fewer than four prior VS sessions (*p* = 0.02). In multivariate analysis, AH was strongly associated with prolonged DE (*p* < 0.001), despite AH patients having a higher BMI (29.8 ± 5.5 vs. 25.2 ± 5.2 kg/m^2^, *p* = 0.001) and being more frequently sedentary (25.5% vs. 13.8%, *p* = 0.07). A trend toward longer DE was observed in patients treated with βBs and ARBs but not with CCBs or ACE inhibitors. Additional independent predictors of longer DE included BMI < 27.5 kg/m^2^ (*p* < 0.001), unicompartmental OA (*p* = 0.02), fewer than four prior VS sessions (*p* = 0.02), and an active lifestyle (*p* = 0.027). **Conclusions:** These findings suggest that antihypertensive treatment may extend the effectiveness of viscosupplementation in knee OA. However, the sample size was insufficient to determine whether specific classes of antihypertensive agents provide greater benefits. Further large-scale, prospective studies are warranted to clarify the potential impact of antihypertensive medications on viscosupplementation outcomes in knee OA.

## 1. Introduction

Knee osteoarthritis (OA) is a highly prevalent musculoskeletal disorder affecting approximately 7.6% of the global population, which translates to nearly 600 million individuals worldwide [1]. Although not directly life-threatening, knee OA significantly impacts patients’ quality of life and can indirectly contribute to increased mortality through its association with obesity and cardiovascular diseases. These two comorbidities are frequently associated with knee OA and are often exacerbated by physical inactivity, disability and the prolonged use of non-steroidal anti-inflammatory drugs (NSAIDs) [2].

Osteoarthritis is characterized by the progressive degeneration of hyaline cartilage, as well as the structural and functional deterioration of the entire joint—including the subchondral bone, synovium, entheses and joint capsule. This process results in chronic pain, stiffness, and impaired mobility, which in severe cases can lead to significant disability. Currently, no treatment exists to regenerate cartilage or halt its degeneration entirely. Conservative management of knee OA relies on a combination of pharmacologic and non-pharmacologic approaches [3,4,5], though none have demonstrated unequivocal efficacy. Among pharmacologic options, viscosupplementation (VS) with intra-articular (IA) hyaluronic acid (HA) injections [6] has shown a clinically relevant effect size (0.63; 95% CrI: 0.39–0.88) [7] and a favorable safety profile, with a relative risk of adverse events comparable to that of saline (1.01; 95% CI: 0.96–1.07; *p* = 0.6) [8], making it a suitable option for elderly patients or those with multiple comorbidities. As a symptomatic treatment for knee OA pain, viscosupplementation is endorsed by several professional societies [3,4,5] for patients who experience inadequate relief with first-line therapies, such as analgesics and NSAIDs. Despite widespread clinical use and positive real-world outcomes [9], the efficacy of viscosupplementation remains debated. As a result, guidelines from organizations such as the Osteoarthritis Research Society International (OARSI) [10] and the American College of Rheumatology (ACR) [11] do not universally endorse viscosupplementation. Eymard et al. identified obesity and radiological severity as independent predictors of a diminished response to viscosupplementation [12], concluding that VS must be used with caution in subjects at risk of treatment failure. Consensus across all major guidelines highlights the importance of an individualized approach to knee OA management, tailoring treatments to each patient’s unique clinical profile and therapeutic response [3,4,5,10,11].

In a previous study [13] that aimed to explore factors influencing the duration of viscosupplementation efficacy in a real-world setting, we identified several factors associated with a longer duration of effectiveness (DoE). The study confirmed the deleterious effect of overweight and advanced stage and showed that sedentary lifestyle and more than four previous viscosupplementations were also predictors of a shorter DoE. Surprisingly, we also identified arterial hypertension as a favorable predictor for a longer treatment efficacy. We therefore questioned the reasons for this observation based on data from the literature. For this, we collected data on the treatments taken by each of the hypertensive patients and performed an analysis comparing patients treated with antihypertensive drugs with those who were not. This paper presents the results of this analysis, which was not initially planned in the study protocol, as well as a detailed analysis of the literature on the subject.

## 2. Patients and Methods

A post hoc analysis was conducted using data from a cross-sectional, single-center study (PRESAGE Study; ClinicalTrials.gov Identifier: NCT04988698), designed to identify predictive factors influencing the duration of viscosupplementation (VS) effectiveness in patients with knee osteoarthritis (OA) under real-world clinical conditions [13]. The study was approved by the French “Comité de Protection des Personnes Sud-EST III” on 21 April 2021 (ID-CRB No. 2021-A00773-38) and adhered to Good Clinical Practice guidelines and the principles outlined in the Declaration of Helsinki.

### 2.1. Patient Selection

The full details of the PRESAGE study protocol have been previously published [13]. In brief, ambulatory adult patients referred to the rheumatology department of the North Franche-Comté Hospital (Belfort, France) from 5 May 2021 to 2 March 2023 were prospectively enrolled if they had received VS for symptomatic knee OA between two months and three years prior to inclusion. The choice of these two timeframes was driven by the necessity to allow for a two-month assessment period to ascertain the effectiveness of VS, as its effects only become apparent over time. After a period of three years, it was deemed that the patient’s self-assessment of functional improvement would lack reliability. As this was a study conducted under “real-life” conditions, there were no exclusion criteria based on age, BMI, or radiological stage. Patients were only excluded if they had cognitive impairments or language barriers preventing comprehension of the study questions or if they declined to provide informed consent.

### 2.2. Study Design

*Data Collection*: During a routine consultation, after obtaining informed consent, investigators systematically collected demographic data and details on current and previous OA treatments, the number of prior VS injections, lifestyle habits, and comorbidities, including related treatments. In the present post hoc analysis, particular attention was retrospectively given to antihypertensive medications, which were categorized into five pharmacological classes: angiotensin-converting enzyme (ACE) inhibitors, calcium channel blockers (CCBs), angiotensin II receptor blockers (ARBs), beta-blockers (βBs), and diuretics.

Knee X-rays were centrally evaluated by an experienced reader to determine the modified Kellgren–Lawrence grade according to Felson [14] and to assess the affected knee compartments.

*Treatment Outcome Assessment:* The primary criterion for treatment effectiveness was self-reported by patients through a standardized questionnaire, which included an evaluation of the duration of effectiveness (DoE), defined as the number of weeks during which VS provided symptom relief. The secondary criteria encompassed patient satisfaction with treatment, patient satisfaction with DoE, and the decrease in painkiller consumption.

### 2.3. Statistical Analysis

In the present post hoc analysis, patients were categorized into two groups: those receiving treatment for arterial hypertension (AH+) and those who were not (AH−). Descriptive statistics were used to summarize the data. Qualitative variables were presented as frequencies and percentages, while quantitative variables were expressed as mean values with standard deviation (SD), along with distribution characteristics (minimum, maximum, and median). Comparisons between the two groups were conducted using appropriate statistical tests: the chi-square test or Fisher’s exact test for categorical variables, and the Mann–Whitney U test for continuous variables. A multivariate regression analysis was performed, incorporating factors that showed a significant association at a 0.2 threshold in previous bivariate analyses. All statistical analyses were conducted using R++© software version 1.4 (Zebrys SAS, Toulouse, France), with an alpha significance level set at 0.05.

## 3. Results

One hundred and five patients (65 women, 40 men) were included. Forty-four subjects had bilateral symptomatic knee OA, requiring injection in 149 knees. One hundred and thirty-three knees were injected using cross-linked HA in a single-injection procedure (five different products). The remaining 16 knees were treated using a three-injection regimen of linear hyaluronic acid (two products). The main patient characteristics are given in Table 1. Patients’ mean age and BMI were 66.1 (range 22–88) years and 27.5 (range 18.3–42.8) kg/m^2^, respectively. The median duration between injection and study visit was 52 weeks (range 25–160; Q1 44-Q3 54). Forty patients (38%) were treated for AH. The average number of antihypertensive molecules was 1.9, ranging from 1 to 4, including CCBs (n = 15), ACE inhibitors (n = 13), diuretics (n = 12), ARBs (n = 7), and βBlockers (n = 6).

The mean DoE of VS was 48.2 + 24.8 weeks in the total population. In bivariate analysis, DoE was unrelated to age, gender, VS dosing regimen, and synovial effusion at the time of injection. DoE was significantly longer in subjects with BMI < 27.5 kg/m^2^ (*p* = 0.002), Kellgren–Lawrence radiological grade <4 (*p* = 0.008), active versus sedentary subjects (*p* = 0.005), unicompartmental involvement (*p* = 0.01), medial tibiofemoral joint space narrowing (*p* = 0.046) and number of previous VS < 4 (*p* = 0.008) (Table 2).

**Figure 1:**
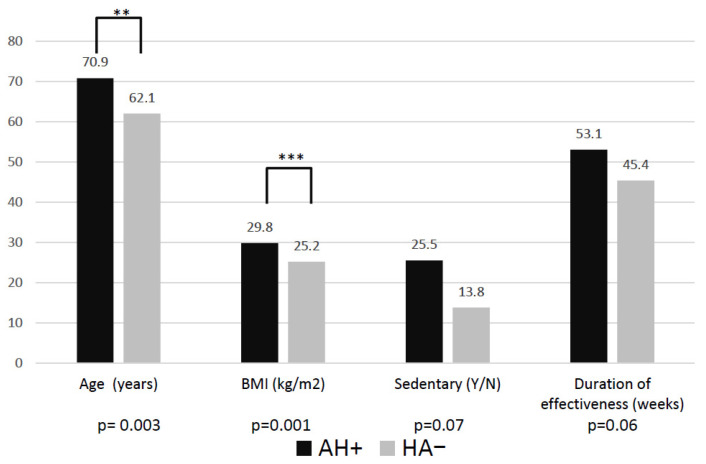
Differences in age, body mass index, sedentary lifestyle and duration of viscosupplementation effectiveness between patients treated (AH+) and not treated for arterial hypertension (AH−) in the bivariate analysis. **: statistically significant *p* = 0.003; ***: statistically significant *p* = 0.001.

In AH+ patients, the average DoE was 53.1 ± 31.3 weeks, compared to 45.4 ± 19.8 weeks in AH− subjects, with a *p*-value of 0.06, approaching statistical significance. There was a trend for longer effectiveness in patients treated with βB (52 vs. 46.5 weeks, *p* = 0.28) and ARBs (54.2 vs. 42.9 weeks; *p* = 0.22), but neither with CCBs (46 vs. 48 weeks; *p* = 0.57) nor ACE inhibitors (46 vs. 48 weeks; *p* = 0.92). Compared to the non-hypertensive group, patients receiving antihypertensive drugs were significantly older (70.9 ± 9.9 versus 62.1 ± 11.4 years; *p* = 0.003), had higher BMI (29.8 ± 5.5 versus 25.2 ± 5.2 kg/m^2^; *p* = 0.001) and more frequently had a sedentary lifestyle (25.5% versus 13.8%; *p* = 0.07) (Figure 1). There were no statistically significant differences observed between the groups in terms of gender, consumption of painkillers, or the viscosupplement used.

In the multivariate analysis, AH+ was strongly associated with a longer DoE (*p* < 0.001), along with four other independent factors: BMI < 27.5 kg/m^2^, unicompartmental knee damage, fewer than four previous viscosupplementations, and an active lifestyle (Table 3).

## 4. Discussion

To our knowledge, this study (while not intended for that purpose) is the first to highlight a strong association between the outcome of knee viscosupplementation—particularly the duration of its effect—and the use of antihypertensive therapy. Despite having a higher BMI and being less physically active, both of which are known to negatively impact the outcomes of hyaluronic acid injections, patients receiving antihypertensive treatment experienced significantly longer-lasting benefits, with symptom relief extending for nearly three months compared with non-hypertensive patients.

It is unlikely that hypertension itself has a positive effect on knee OA symptoms. Indeed, observational studies have demonstrated an association between systolic blood pressure and pain intensity in knee osteoarthritis, independent of radiographic severity [15].

Therefore, the most plausible hypothesis is that certain antihypertensive medications may exert beneficial effects. Our findings are consistent with existing literature suggesting potential chondroprotective effects of specific antihypertensive agents. A trend toward prolonged efficacy was observed in patients treated with β-blockers and ARBs. In preclinical studies, ARBs and ACEi protect cartilage by blocking the AT1 receptor and the renin-angiotensin system (RAS), thereby limiting oxidative stress, enzymatic degradation, and fibrosis, while positively modulating chondroprotective cellular pathways [16,17,18,19,20]. Our findings suggest that β-blocker use may be associated with improved durability of viscosupplementation, in agreement with several studies showing their beneficial effect on OA pain. β-blockers have been associated with reduced pain and lower opioid consumption in patients with OA compared with other antihypertensive therapies, including in a prospective cohort study with a four-year follow-up, suggesting a potential analgesic effect possibly mediated by central mechanisms via the parasympathetic nervous system, independent of blood pressure reduction [21,22]. Similar results were obtained in 111,718 β-blocker users who were compared with unexposed controls. β-blocker therapy was associated with reduced cumulative risks of consultations for knee OA, knee pain, and hip pain, with hazard ratios (95% confidence intervals) of 0.90 (0.83–0.98), 0.88 (0.83–0.92), and 0.85 (0.79–0.90), respectively [23]. Nevertheless, other studies, including one based on Mendelian randomization [24], did not demonstrate any association between β-blockers and osteoarthritis-related pain [24,25].

In contrast, patients treated with CCBs appear to experience greater pain and higher rates of joint replacement surgery [15]. This effect may result from reduced chondrocyte proliferation [26] or from muscular effects mediated through glucose transporters [27], but remains controversial due to conflicting results [28,29,30,31]. These findings may explain why, in our cohort, patients who were administered CCBs did not demonstrate a prolonged DoE when compared to those who were not treated for hypertension.

Beyond an analgesic effect via OA management, the prolonged duration of viscosupplementation efficacy observed in our study may also result from more specific interactions between antihypertensive treatments and the biological mechanisms of HA. HA is not limited to a simple mechanical lubricating effect but also acts as a biological modulator of OA by inhibiting pro-inflammatory cytokines (IL-1β, TNF-α), reducing metalloproteinase activity, and stimulating endogenous HA synthesis [32,33,34,35,36]. In this context, antihypertensive treatments, particularly renin–angiotensin system (RAS) inhibitors and beta-blockers, may create a more favorable intra-articular environment for the prolonged action of injected HA by reducing synovial inflammation, microvascular dysfunction and neuro-inflammatory processes, which are parameters known to predict a worse outcome [37]. Indeed, beyond the circulating RAS involved in blood pressure regulation, an inducible local (‘tissue’) RAS has been described within the joint (notably synovium and chondrocytes), where it can act in an autocrine/paracrine manner and contribute to synovial inflammation and cartilage catabolism through the Angiotensin II—AT1 receptor axis. [38,39]. In this setting, ACEi/ARBs may modulate the intra-articular milieu not only by attenuating AT1 signaling, but also by favoring the counter-regulatory ACE2/Angiotensin-1–7/Mas receptor pathway, which is associated with anti-inflammatory and anti-fibrotic effects in experimental arthritic conditions [40,41]. This potential “rebalancing” could, in turn, contribute to a more permissive environment for a prolonged symptomatic response after IA-HA. This pharmacodynamic synergy could explain the trend for prolonged efficacy observed in our patients treated with ARBs (54.2 vs. 42.9 weeks; *p* = 0.22). Such a result was not found in subjects receiving ACE inhibitors (46 vs. 48 weeks; *p* = 0.92). However, as most patients were receiving multiple antihypertensive drugs, and, for most patients, we were unable to obtain the date of initiation of antihypertensive treatment, definitive conclusions regarding the benefits of any specific therapeutic class cannot be drawn.

The main strength of this study lies in the identification of a modifiable clinical factor, treated hypertension, never explored in the context of viscosupplementation, as well as in the analysis of duration of efficacy as a clinically relevant outcome. Multivariate adjustment accounting for recognized predictors of response to viscosupplementation (age, BMI, physical activity, and clinical severity) strengthens the robustness of the observed association, despite our inability to analyze individual antihypertensive therapies separately.

Nevertheless, several limitations should be acknowledged, including the post hoc and single-center design of the study. The present study was of an observational and pragmatic nature, conducted in close proximity to real-life settings. The outcomes of viscosupplementation were assessed using daily practice tools, which contain a high degree of subjectivity. The primary bias is attributable to the fact that, at the time the protocol was designed, it was not anticipated that HTA would exert a substantial influence on the results. Consequently, the collection of blood pressure data was not undertaken, thereby precluding the confirmation of the absence of hypertension in the control group. A salient potential bias, which may offer a partial explanation for our findings and cannot be disregarded, is that patients treated with antihypertensive agents may undergo more frequent primary care monitoring and receive more regular lifestyle counseling. However, given their higher BMI and lower physical activity levels, this effect does not appear to have had a major impact on our results. Another factor that has not been taken into account is the presence/absence of crystal deposition disease, particularly calcium pyrophosphate, which can contribute significantly to the progression of osteoarthritis and DoE. Finally, the paucity of data on the natural progression of osteoarthritis in hypertensive patients prevents us from formally attributing our results solely to the effect of viscosupplementation.

A number of studies have explored the relationship between hypertension and the clinical expression of knee OA. However, data specifically addressing disease progression remains limited. In a large cohort from the Osteoarthritis Initiative (n = 2906), elevated blood pressure was significantly but moderately associated with weight-bearing knee pain progression (OR = 1.09, 95% CI 1.02–1.17), suggesting a possible vascular contribution to symptomatic worsening [42]. Furthermore, stage 2 hypertension has been associated with increased knee pain severity, after adjustment for confounders [43]. Cross-sectional evidence also indicates that hypertensive women have higher odds of late-stage radiographic OA than normotensive individuals, although longitudinal structural data are scarce [44]. Importantly, recent longitudinal analyses of comorbidities have identified diabetes and kidney disease as predictors of symptomatic progression, but not hypertension [45]. This highlights the current uncertainty regarding its independent role. While hypertension is generally linked to a higher symptom load and potentially more advanced structural disease, there is a lack of robust prospective studies specifically evaluating radiographic progression in hypertensive versus normotensive patients.

## 5. Conclusions

In summary, this observational clinical trial, while primarily not designed for assessing the role of antihypertension in patients treated with IA-HA for knee OA, suggests that antihypertensive treatment may extend the effectiveness of viscosupplementation. However, the sample size was insufficient to determine whether specific classes of antihypertensive agents provide greater benefits. Therefore, this unexpected association between antihypertensive drug use and viscosupplementation outcomes should be interpreted cautiously, as it raises two important questions: whether the association is genuine and, if confirmed, what mechanisms underlie it. Many uncertainties remain regarding the tissue effects of antihypertensive drugs on joints, such as the unknown effects on the vascularization of the synovial membrane and adipose tissue. Further large-scale, prospective studies are warranted to clarify the potential impact of antihypertensive medications on viscosupplementation outcomes in knee OA.

## Figures and Tables

**Table 1:** Main patient characteristics at time of injection (n = 105).

**Age** ± SD [range] **(Years)**	66.1 ± 13.2 [22–88]
**Gender** N (%)	
Female	66 (62%)
Male	39 (38%)
**Body mass index (kg/m^2^)**	
Median	26.8
Mean ± SD [range]	27.5 ± 5.55 [18.3–43.7]
**Time since viscosupplementation (weeks)**	
Median [range]	52 [25–160]
Mean ± SD	54.4 ± 24.4
**Professional status** N (%)	
Working	35 (33%)
Unemployed	10 (10%)
Retired	60 (57%)
**Sport practice** N (%)	
Yes	46 (43.8%)
No	59 (56.2%)
**Antihypertensive drugs** N (%)	
Yes	40 (38%)
No	65 (62%)

**Table 2:** Predictors of the duration of effectiveness of viscosupplementation: bivariate analysis.

	DoE (Weeks) (mean ± SD) [Range]	CI 95%	*p*-Value
**BMI** kg/m^2^			
<27.5	53.4 ± 29.7 [4–156]	47.0–59.9	0.002
>27.5	41.16 ± 14.36 [0–65]	38.0–45.0	
**Physical activity**			
Active	50.3 ± 25.5 [0–156]	45.7–54.9	0.005
Sedentary	38.7 ± 19.0 [16–80]	31.2–46.3	
**Kellgren-Lawrence grade**			
Grade < 4	51.8 ± 26.6 [0–156]	46.6–56.9	0.008
Grade 4	40.0 ± 17.9 [4–100]	34.7–45.4	
**Number of viscosupplementations**			
1–3	51.9 ± 29.7 [0–156]	45.7–58.1	0.008
>3	42.5 ± 12.3 [16–73]	39.2–45.7	
**Treatment for arterial hypertension**			
No (AH−)	45.4 ± 19.8 [0–124]	41.3–49.4	0.07
Yes (AH+)	53.1 ± 31.3 [16–156]	44.6–61.5	
**Number of involved compartments**			
1	52.5 ± 27.3 [0–156]	46.7–58.2	0.01
>1	41.6 ± 19.7 [4–109]	36.4–46.8	
**Knee strain**			
High	48 ± 25.98 [0–156]	37.2–59.1	0.02
Normal/moderate	50.31 ± 25.14 [4–155]	45.4–55.2	
Low	38.5 ± 20.425 [16–80]	29.4–47.6	

DE: Duration of Effectiveness; BMI: Body Mass Index.

**Table 3:** Predictors of the duration of effectiveness of viscosupplementation: multivariate analysis (coefficient of determination: R = 0.509; R^2^ = 0.259).

Predictors	Estimation	Standard Error	t	*p*
**Intercept**	59.75	3.38	17.6	**<0.001**
**BMI**:				
>27.5 versus <27.5	−14.92	4.15	−3.6	**<0.001**
**Number of involved compartments**				
**>2** versus 1	−9.13	3.96	−2.3	**0.023**
**K-L grade:**				
Grade 4 versus Grade < 4	−5.49	4.46	−1.2	0.220
**Number of VS:**				
>3 versus 1 to 3	−9.35	3.94	−2.3	**0.019**
**Antihypertensive treatment**				
Yes (AH+) versus No (AH−)	16.39	4.23	3.88	**<0.001**
**Physical activity:**				
Sedentary versus Active	−11.75	5.25	−2.2	**0.027**

## Data Availability

Data are available at the Clinical Research Unit of Hôpital Nord Franche-Comté (URC HNFC), Trevenans, France.
